# Predictors of Delayed Recovery in Japanese Patients With Whiplash-Associated Disorders: The Role of Initial Catastrophizing and Disability Levels

**DOI:** 10.7759/cureus.66435

**Published:** 2024-08-08

**Authors:** Yuh Yamashita, Haruki Kogo, Tadatoshi Inoue, Toshio Higashi

**Affiliations:** 1 Graduate School of Biomedical Sciences, Nagasaki University, Nagasaki, JPN; 2 Department of Rehabilitation, Morinaga Orthopedic Clinic, Saga, JPN; 3 Faculty of Rehabilitation Sciences, Reiwa Health Sciences University, Fukuoka, JPN; 4 Department of Rehabilitation Major in Occupational Therapy, Heisei College of Health Sciences, Gifu, JPN

**Keywords:** treatment duration, pain management, whiplash-associated disorders, disability, catastrophizing

## Abstract

Background

Whiplash-associated disorders are sequelae of traffic accidents that frequently result in sustained pain and disability due to a broader spectrum of symptoms than typical neck pain. Several studies have used the length of time from injury to the completion of insurance claims as a measure of recovery time for patients with whiplash-associated disorders. However, studies on the initial factors in patients whose treatment exceeds 90 days are lacking. Therefore, this study aimed to identify key factors predicting prolonged treatment duration in Japanese patients with whiplash-associated disorders.

Methodology

We included 103 outpatients who presented with neck pain after a motor vehicle accident. During their initial visits, various factors were comprehensively assessed, including pain intensity, Neck Disability Index (NDI), six items of the Pain Catastrophizing Scale (PCS-6), a short version of the Tampa Scale of Kinesiophobia, the Injustice Experience Questionnaire, cervical range of motion, and radiographic findings. Patients were categorized into “early recovery” or “delayed recovery” groups based on the time elapsed between the first assessment and the end of the treatment period. Logistic regression analysis identified cut-off values from receiver operating characteristic curves to help identify factors contributing to delays in the recovery process.

Results

Analysis showed that initial NDI and PCS-6 scores of ≥35% and ≥12, respectively, were significant predictors of delayed recovery, increasing the odds of delay by factors of 3.19 and 4.46, respectively.

Conclusions

Our findings may aid in appropriate clinical decision-making and lead to interventions to minimize the negative impact of prolonged treatment duration on patient recovery.

## Introduction

Neck pain is a prevalent symptom worldwide [1], with approximately 290 million individuals experiencing chronic neck pain [1]. Whiplash-associated disorders (WADs) are particularly noted as sequelae of traffic accidents and frequently lead to sustained pain and disability due to a broader spectrum of symptoms than typical neck pain [2]. The severity of the traffic collision does not consistently predict recovery in patients with WAD [3,4]. Psychological factors, including perceived symptoms, anxiety, depression, and catastrophizing, wield a more significant impact in determining prognosis than mechanical injury-related factors [5]. Furthermore, many patients with WADs are injured due to third-party actions, and the subsequent compensation process, encompassing negotiations and legal disputes, may intricately shape their recovery trajectory [6].

The persistent absence of recovery from whiplash injuries imposes significant personal, economic, and societal burdens, thereby underscoring the need for targeted interventions to decrease the number and percentage of patients with WADs experiencing chronic conditions. Several studies have used the length of time from injury to the completion of insurance claims as a measure of recovery time for patients with WADs [7-9]. Claim finalization signifies the cessation of treatment, attainment of maximal medical improvement, or end-of-income replacement benefits. Given that the symptoms of WAD frequently diminish rapidly at the outset, numerous investigations have concentrated on therapeutic measures within the first 90 days post-injury [10,11]. However, symptoms that persist beyond 90 days post-injury frequently lead to chronic conditions [12].

Previous studies have established a correlation between the time required to settle a claim and initial functional disability, psychological distress, and compensation-related legal issues [7-9]. However, research on the initial factors in patients whose treatment exceeds 90 days is lacking. Identifying these risk factors at an early stage can help healthcare professionals conduct prompt evaluations and inform insurance claim strategies. Therefore, this study aimed to identify the predictive factors and their discriminative precision for the extended timeframe between injury occurrence and the resolution of insurance claims in patients with WADs during the initial evaluation.

## Materials and methods

Study design and participants

This prospective study enrolled 191 Japanese patients diagnosed with WAD from primary orthopedic clinics between June 2018 and June 2022. The study was conducted at Morinaga Orthopedic Clinic in Saga, Japan, and Rokuto Orthopedic Clinic in Okinawa, Japan. The inclusion criteria were WAD onset resulting from a vehicle collision, patient age between 20 and 80 years, onset occurring within 14 days before clinic visitation, classification as Grade I or II according to the Quebec Taskforce on WAD [13], and the patient having sought assistance through a single walk-in visit to the primary clinic. The exclusion criteria included a history of cervical spine trauma or surgery, severe acute post-traumatic stress disorder, any central nervous system disease, a history of severe mental illness or dementia, patients classified as Grade Ⅲ or higher according to the Quebec Taskforce on WAD [13], and those for whom radiographic image data could not be obtained. All participants received standardized care comprising behavioral strategies, educational resources, self-care guidelines, and necessary medications [14]. This research adhered to the ethical principles outlined in the Declaration of Helsinki. Ethics approval was obtained from the Institutional Ethics Committee of Nishikyushu University (approval number: H30-3, approved on June 18, 2018). All participants provided written informed consent before the study.

Procedures

Demographic data of the participants, including age, sex, height, and weight, were evaluated. Additionally, Treatment duration, neck-specific disability levels, pain intensity, catastrophizing, fear of movement, and perceived injustice were assessed. Physical function was examined by measuring the range of motion of the cervical joints in three directions, and structural assessment was performed by visually analyzing simple radiographic images. After collecting all initial evaluation data, we longitudinally tracked the period from the start of treatment to the closure of the insurance claim using patient medical records.

Classification by Treatment Duration for Whiplash-Associated Disorders

The duration of treatment is frequently used as an outcome measure in cohort studies of WADs. In this study, we categorized participants into two groups based on the duration from the start of treatment to the completion of insurance claims: the early recovery group, which included those who completed treatment in under 90 days, and the delayed recovery group, which included those whose treatment lasted longer than 90 days.

Neck-Specific Disability

The Neck Disability Index (NDI) was used to assess neck-specific disability [15,16]. The NDI is internationally recognized as the most widely used self-report instrument for cervical pain and comprises the following 10 items: seven related to activities of daily living, two concerning pain level, and one regarding concentration. Each item is scored from 0 to 5, resulting in a cumulative score ranging from 0 to 50, with higher scores indicating more significant disability. Consistent with previous research, the total NDI score was divided by the number of items answered and expressed as a percentage [16].

Pain Intensity

The Visual Analog Scale was used to assess pain intensity. Specifically, the average pain intensity over the past seven days was rated on a 100-mm scale, with “no pain” and “maximum imaginable pain” at the left and right ends, respectively, ranging from 0 to 100 mm.

Catastrophizing

The degree of pain-related catastrophizing was assessed using the short version of the Pain Catastrophizing Scale-6 (PCS-6) [17]. The PCS-6 comprises the following three subscales: two items each for “rumination,” “helplessness,” and “magnification.” Each item was rated on a scale of 0-4, with a total score of 0-24 and higher scores indicating more catastrophic thinking [17].

Fear of Movement

The degree of pain-related fear of movement was assessed using the short version of the Tampa Scale for Kinesiophobia (TSK-11) [18]. Each item was rated on a scale of 1-4, with total scores ranging from 11 to 44 and higher scores indicating stronger motor fear beliefs [18].

Perceived Injustice

Perceived injustice was measured using the Injustice Experience Questionnaire [19]. This comprehensive 12-item survey is designed to assess the intensity of an individual’s sense that their present circumstances are unjust. The scoring system ranges from 0 to 48, with elevated scores indicative of heightened perceptions of unfairness [19].

Active Cervical Range of Motion

The active cervical range of motion (ACROM) was measured using a universal goniometer, which is the most commonly used device for measuring joint range of motion and is reliable for measuring the automatic range of motion of the cervical spine [20]. ACROM was measured two times each in the order of flexion, extension, right lateral flexion, left lateral flexion, right rotation, and left rotation, and the maximum angle in each direction was recorded as the representative value. For lateral flexion and rotation ACROM, the sum of the left and right values was used in the analysis.

Radiographical Findings

Vertebral degenerative findings were measured according to the degenerative index proposed by Kumagai et al. [21]. Degenerative changes in the disc space, endplate sclerosis, and the presence of anterior and posterior osteophytes on lateral radiographs were graded using a four-point scale ranging from 0 to 3 points for each parameter. The cumulative score for these factors across the six vertebrae from C2 to C7 was employed for subsequent analysis [21]. Additionally, the rate of spinal canal stenosis was analyzed using the Torg-Pavlov ratio [22]. The anterior-posterior diameter of the vertebrae up to C2-7 in the lateral radiographs divided by that of the spinal canal was used for analysis. The minimum values of the Torg-Pavlov ratio at C2-3, C3-4, C4-5, C5-6, and C6-7 were used for analysis. Sagittal plane alignment was assessed using Harrison’s posterior tangent method; the sagittal plane kyphosis angle was calculated from the tangent of the posterior edges of the C2 and C7 vertebral bodies [23].

Statistical analysis

Of the 191 participants, complete data from 103 patients, with no missing values, were included in the analysis. Univariate logistic regression analysis was conducted to examine the association between the presence or absence of delayed recovery and each variable. Variables found to have significant associations were regarded as potential predictors of prolongation. Based on the receiver operating characteristic (ROC) curve analysis for these predictors, the cutoff value was set at the point where the sum of sensitivity and specificity was most significant when assuming a positive delayed recovery. We also conducted a multivariate logistic regression analysis using a variable reduction method. The independent variables in this analysis were dichotomized according to specified cutoff values, aiming to pinpoint the most influential predictors linked with delayed recovery. To analyze the discriminative accuracy of the predictors, the sensitivity, specificity, positive predictive value (PPV), and negative predictive value (NPV) were calculated by comparing the predicted and measured values. Sensitivity was calculated as the proportion of true positives identified by the model, while specificity was the proportion of true negatives. PPV was the proportion of positive test results that were true positives, and NPV was the proportion of negative test results that were true negatives. Statistical analyses were performed using EZR (version 1.35, Saitama Medical Center, Jichi Medical University), which is the user interface of R (R Foundation for Statistical Computing). The statistical significance level was set at 5% (p < 0.05).

## Results

Table 1 presents the characteristics of the participants. The mean duration of treatment was 81.4 ± 52.9 days, and the mean age of the participants was 46.1 ± 14.8 years, of whom 63 (61.2%) were female and 86 (83.5%) were employed. Overall, 58 (56.3%) patients were in the early recovery group (<90 days), and 45 (43.7%) were in the delayed recovery group.

**Table 1:** Participant characteristics. Data are reported as the mean ± standard deviation or n (%). BMI: body mass index

Characteristic	All cases (N = 103)
Age (year)	46.1 ± 14.8
Sex (female)	63 (61.2%)
BMI (kg/m^2^)	23.1 ± 3.8
Paid jobs (yes)	86 (83.5%)
Treatment duration (days)	81.4 ± 52.9

Table 2 shows the results of the univariate logistic regression analysis. Univariate logistic regression analysis showed that increased NDI, PCS-6, and TSK-11 scores and decreased gyration cervical range of motion (CROM) significantly increased the odds of delayed recovery; however, no significant associations were found for other variables.

**Table 2:** The results of univariate logistic regression analysis (early recovery group, n = 58 vs. delayed recovery group, n = 45). Bold type indicates statistical significance (p < 0.05). Early recovery group: patients with a recovery period of less than 90 days. Delayed recovery group: patients with a recovery period of 90 days or more. BMI: body mass index; NDI: Neck Disability Index; VAS: Visual Analog Scale; PCS-6: 6-item short version of the Pain Catastrophizing Scale; TSK-11: 11-item short version of the Tampa scale for kinesiophobia; IEQ: Injustice Experience Questionnaire; ACROM: active cervical range of motion; TPR: Torg-Pavlov ratio; OR: odds ratio; CI: confidence interval; SE: standard error.

Factor	B	SE	P-value	OR	95% CI
Characteristics					
Age	0.012	0.014	0.347	1.01	0.99−1.04
Sex (female)	0.087	0.407	0.831	1.09	0.49−2.42
BMI	0.035	0.053	0.505	1.04	0.93−1.15
Paid job (yes)	-0.733	0.539	0.174	0.48	0.17−1.38
Specific questionnaire					
NDI	0.051	0.016	<0.001	1.05	1.02−1.09
VAS	0.005	0.009	0.565	1.00	0.98−1.02
PCS-6	0.135	0.037	<0.001	1.14	1.06−1.23
TSK-11	0.127	0.039	0.001	1.14	1.05−1.23
IEQ	0.057	0.029	0.051	1.06	1.00−1.12
ACROM					
Flexion	-0.019	0.019	0.332	0.98	0.95−1.02
Extension	-0.001	0.020	0.974	0.98	0.96−1.04
Lateral bending	0.019	0.017	0.288	5.39	0.98−1.05
Rotation	-0.020	0.008	0.031	0.98	0.97−0.99
Radiographic findings					
Degenerative Index	0.036	0.031	0.255	1.04	0.97−1.10
TPR	-1.089	1.325	0.411	0.34	0.02−4.51
Sagittal plane alignment	-0.011	0.016	0.491	0.99	0.96−1.02

Table 3 shows the results of the ROC analysis of the four variables that displayed significant associations in the univariate logistic regression analysis. ROC curve analysis was used to determine the optimal cutoff values for predictor variables based on the highest sum of sensitivity and specificity. Specifically, thresholds of 35% for NDI (sensitivity: 51.1%, specificity: 81.0%), 12 points for PCS-6 (sensitivity: 62.2%, specificity: 77.6%), 25 points for TSK-11 (sensitivity: 62.2%, specificity: 72.4%), and a gyration CROM of 135° (sensitivity: 82.2%, specificity: 32.8%) were identified as the cutoff points with the most significant discriminatory power.

**Table 3:** ROC analysis for predictors extracted by univariate logistic regression analysis. NDI: Neck Disability Index; PCS-6: 6-item short version of the Pain Catastrophizing Scale; TSK-11: 11-item short version of the Tampa scale for kinesiophobia; ACROM: active cervical range of motion; ROC: receiver operating characteristic; CI: confidence interval; AUC: area under the ROC curve

	Cutoff value	Sensitivity (%)	Specificity (%)	AUC (95% CI)
NDI	≥35%	51.1	81.0	0.69 (0.59−0.79)
PCS-6	≥12 points	62.2	77.6	0.72 (0.62−0.82)
TSK-11	≥25 points	62.2	72.4	0.69 (0.59−0.79)
ACROM (rotation)	≤135°	82.2	32.8	0.63 (0.52−0.74)

Table 4 shows the results of backward stepwise multiple regression analysis, which indicated that NDI and PCS-6 were the primary determinants of delayed recovery. Specifically, an NDI ≥35% increased the odds of delayed recovery by a factor of 3.19 (95% confidence interval: 1.24-8.17), and a PCS-6 ≥12 increased the odds by a factor of 4.46 (95% confidence interval: 1.82-11.00).

**Table 4:** Multivariate logistic regression analysis for predictor variables dichotomized based on cutoff values. NDI: Neck Disability Index; PCS-6, 6-item short version of the Pain Catastrophizing Scale; CI: confidence interval; SE: standard error; adj.OR: adjusted odds ratio

	B	SE	P-value	adj.OR	95% CI
NDI ≥35	1.16	0.48	0.012	3.19	1.24−8.17
PCS-6 ≥12	1.49	0.46	0.001	4.46	1.82−11.00
(Intercept)	-1.26	0.32	<0.001		

Table 5 presents the accuracy statistics for the predictors identified in the multivariate logistic regression analysis. When the two predictors were analyzed separately, the discrimination accuracy yielded the following results: PCS-6 had a sensitivity, specificity, PPV, and NPV of 62.2%, 77.6%, 68.3%, and 72.6%, respectively; NDI had a sensitivity, specificity, PPV, and NPV of 51.1%, 81.0%, 67.6%, and 68.1%, respectively. However, when the two predictors were combined for analysis, they had a sensitivity, specificity, PPV, and NPV of 33.3%, 89.7%, 71.4%, and 63.3%, respectively.

**Table 5:** Accuracy statistics (and 95% CI) for predictors of delayed recovery extracted by multivariate logistic regression analysis. Prevalence, 43.7%. NDI: Neck Disability Index; PCS-6: 6-item short version of the Pain Catastrophizing Scale; CI: confidence interval; PPV: positive predictive value; NPV: negative predictive value

	Positivity rate (%)	Sensitivity (%)	Specificity (%)	PPV (%)	NPV (%)
PCS-6 ≥12	39.8 (30.3−49.9)	62.2 (46.5−76.2)	77.6 (64.7−87.5)	68.3 (51.9−81.9)	72.6 (59.8−83.1)
NDI ≥35	33.0 (24.1−43.0)	51.1 (35.8−66.3)	81.0 (68.6−90.1)	67.6 (49.5−82.6)	68.1 (55.8−78.8)
NDI ≥35 and PCS-6 ≥12	20.4 (13.1−29.5)	33.3 (20.0−29.0)	89.7 (78.8−96.1)	71.4 (47.8−88.7)	63.3 (52.0−74.2)

## Discussion

This study provides preliminary insights into the factors that can help predict treatment duration among individuals with WAD, along with initial evidence supporting their predictive accuracy. These findings suggest that high levels of catastrophic thinking (PCS-6 ≥12) and impaired ability (NDI ≥35) at initial diagnosis are predictors of delayed recovery in Japanese patients with WAD, and the cutoff values determined through ROC analysis have acceptable discriminatory accuracy. In addition, early catastrophizing was more strongly associated with delayed recovery than self-reported disability. Patients involved in road traffic accidents typically experience higher levels of psychological distress than those with other musculoskeletal disorders, and these psychological factors play a substantial role in shaping the trajectory of future recovery [5]. Catastrophizing may promote psychological distress associated with pain [24] and have detrimental consequences for the future physical and mental health of patients with chronic pain [25]. A previous cohort study of patients with WAD showed that helplessness, a subscale of the PCS, was the greatest predictive factor for disability levels and poor mental and physical health [25].

The results of this study showed that a PCS-6 score of ≥12 at the initial visit increased the odds of prolonged recovery by 4.46 times. The first evidence supporting this finding is that catastrophizing is associated with expectations of increased pain and emotional distress [25]. Expectations of adverse outcomes have been proposed to detrimentally impact behavior and performance by undermining the effort and motivational resources necessary to assist patients in achieving specific goals [26]. In a previous extensive study of Japanese patients [27], the presence of not only severe subjective symptoms but also low expectations for future recovery was identified as a potential risk factor for prolonged treatment of patients with WAD. Specifically, severe catastrophizing in acute WAD may prolong claim closure by inhibiting patients’ proactive behavior toward recovery through distorted cognitive processes.

Second, pain catastrophizing is associated with changes in the descending pain modulatory network of the central nervous system [28]. Recent brain imaging studies have suggested an association between pain catastrophizing, cortical function, and organic reorganization [29]. An extreme increase in attention to pain due to pain catastrophizing may alter the central nervous system processing mechanism of pain, thereby contributing to the chronic trajectory. However, it is important to note that the current study uses the treatment duration as an outcome for recovery and, therefore, does not directly demonstrate an association between catastrophizing and pain or disability outcomes.

Accuracy analysis in this study revealed that a PCS score ≥12 exhibited the highest sensitivity (62.2%) and NPV (72.6%) when examined independently, suggesting that using the PCS cutoff value during the initial assessment can help predict relatively early recovery (within three months) from injury to treatment completion. Early aggressive treatment of patients experiencing neck pain may increase their expectations of poor outcomes, which, in turn, may lead to prolonged pain [30]. Early triage of patients with WAD using the PCS-6 cutoff value suggests the possibility of early identification of patients at low psychological risk for pain. This helps clinicians make optimal treatment decisions for patients with acute WAD by reducing the requirement for unnecessary information and interventions associated with treatment. Additionally, our study revealed that when combined, PCS-6 ≥12 and NDI ≥35 exhibited a higher PPV compared to analyzing each variable alone. The combination of these endpoints had a high specificity (89.7%) for patients with WAD in the early stages of the disease, which may enable the early identification of patients at high risk for prolonged treatment duration. However, it is necessary to note that these combinations had a low sensitivity of 33.3%, which could lead to erroneous identification of a positive patient as negative. Therefore, the effectiveness of clinical decision-making using these cutoff values should be further validated in future randomized controlled trials.

Nevertheless, this study has some limitations that must be considered. First, extracting predictors from a relatively small sample may suffer from inadequate statistical power, and demographic bias could be present in the population. Second, the results obtained in this study may lack predictive validity, and it may be premature to utilize the predictive value in clinical practice. Research evaluating predictors of treatment duration is still in its nascent stage, necessitating further multicenter studies.

## Conclusions

This study provides new information regarding the early factors influencing prolonged treatment duration in patients with WAD. Our findings suggest that using the NDI and the PCS-6 in the initial assessment of patients with WAD may help predict the risk of prolonged treatment duration. The insights gained from this study are valuable for appropriate clinical decision-making by healthcare providers, potentially leading to interventions that minimize negative impacts on patient recovery.
